# The Relationship between Sleep Duration and Stroke Risk: The Mediating Role of Physical Activity

**DOI:** 10.3390/brainsci12050601

**Published:** 2022-05-05

**Authors:** Xingyue Liu, Juhua Zhang, Yanmei Wang, Changlian Lu, Xuefeng Gu, Guoqing Wan, Peng Zhang

**Affiliations:** 1 Graduate School, Shanghai University of Traditional Chinese Medicine, Shanghai 201203, China; mumaoxy@163.com; 2 Department of Social Medicine and Health Career Management, Fudan University, Shanghai 200433, China; hua73825@126.com; 3 Research Center, Shanghai Pudong Health Development Research Institute, Shanghai 200129, China; 4 Department of Integrated Traditional Chinese and Western Medicine, Shanghai University of Medicine and Health Sciences Affiliated Zhoupu Hospital, Shanghai 201318, China; 5 Department of Nursing, Second Military Medical University Affiliated Shanghai Gongli Hospital, Shanghai 200135, China; wangym0101@126.com; 6 Collaborative Research Center, Shanghai University of Medicine and Health Sciences, Shanghai 201318, China; lvcl@sumhs.edu.cn (C.L.); guxf@sumhs.edu.cn (X.G.); wangq@sumhs.edu.cn (G.W.); 7 School of Clinical Medicine, Shanghai University of Medicine & Health Sciences, Shanghai 201318, China

**Keywords:** stroke risk factors, sleep, physical activity, mediation analysis, suburban residents

## Abstract

Background: This study aimed to investigate the mediating effect of physical activity (PA) on the relationship between average sleep duration and risk of stroke in suburban residents without stroke. Methods: A cross-sectional study was executed, and participants were recruited through a multistage, stratified, probability-proportional-to-size sampling method in this research. The stroke risk was measured using a risk assessment form for a high-risk stroke population. The PA score was calculated by the Physical Activity Rating Scale-3 (PARS-3). The average sleep duration was calculated by adding up night sleep and afternoon nap durations. A multiple linear regression analysis was conducted to identify the association between stroke risk, average sleep duration, and PA. The direct and indirect effects of average sleep duration on stroke risk were analyzed by using the PA in a mediation framework. Results: A total of 5312 suburban residents (average: 54.96 ± 12.21 years, 2970 women) participated in the study. After adjusting for covariates, relatively inappropriate sleep duration (<7 h/>8 h~9 h/>9 h) and stroke risk were significantly associated, compared with the moderate average sleep duration (7~8 h) (β = 0.038, 95% CI: 0.024~0.128; β = 0.078, 95% CI: 0.128~0.250; β = 0.150, 95% CI: 0.390~0.549). The PA total score (indirect effect ab = 0.013, 95% CI: 0.003~0.022) partially mediated the relationship between the long average sleep duration and stroke risk, in which the activity intensity (ab = −0.015, 95% CI: −0.021~−0.008), the activity duration (ab = 0.043, 95% CI: 0.029~0.058), and the activity frequency (ab = 0.012, 95% CI: 0.004~0.020; ab = 0.037, 95% CI: 0.026~0.050) all played a mediating role in the different sleep duration. Conclusions: A significant relationship between a long average sleep duration and stroke risk factors among people without stroke was found in this study. The PA and its components partially mediated the association between a long average sleep duration and stroke risk. Suitable prevention methods and interventions for PA and sleep may reduce the risk of stroke.

## 1. Introduction

Stroke is defined as the sudden start of a neurologic impairment caused by a disruption in the blood flow to the brain that lasts more than 24 h. It is a prominent cause of death and disability across the world, with high morbidity, disability, mortality, recurrence, and economic burden [1]. Between 1990 and 2019, the absolute number of incident strokes grew by 70% (67.0–73.0), prevalent strokes increased by 85% (83.0–88.0), stroke mortality climbed by 43% (31.0–55.0), and DALYs attributable to stroke increased by 32% (22.0–42.0) [2]. Stroke is also the leading cause of death and disability in adults in China [3,4]. In recent years, China’s stroke disability and fatality rates have continued to climb, posing a serious threat to patients’ families and society [5,6,7]. Given the serious consequences and accompanying burden, stroke prevention is a critical aspect of the Chinese national health policy.

Sleep deprivation and physical inactivity are frequent in adults, and both can increase the occurrence of stroke [8]. Stroke risk factors include advanced age, hypertension, diabetes, high cholesterol, cigarette smoking, atrial fibrillation, etc. [9]. Recently, the quality and duration of sleep have been associated with stroke, and sleep disturbances and sleep-related disorders exacerbate the above-mentioned risk factors [10,11], while sleep duration has long been thought to be a major risk factor for stroke. Sleep is a crucial natural function in our daily life, accounting for one-third of human life, and the quality and length of sleep are closely related to our health status. Suboptimal sleep duration has been significantly associated with stroke risk factors in recent decades [10,11,12,13]. Several studies showed a curvilinear link between sleep duration and stroke occurrence; however, there are differences in the literature supporting this correlation, which may be J-shaped [14], U-shaped [15], or neither pattern [16].

Physical activity (PA), as a modifiable risk factor, has been increasingly noted for its positive effects on brain health in recent years. Existing studies demonstrated that PA not only improves cardiovascular health but also slows cognitive decline and reduces the incidence of dementia [17,18,19]. Moreover, multiple reviews have pointed out that physically active people are less likely to suffer a stroke than sedentary people [20,21]. Higher levels of prestroke PA are linked to a lower risk of stroke severity [22]. PA reduces the risk of stroke by decreasing subclinical risk factors such as poor endothelial function, high cholesterol, an increased BMI, blood clots, and limited blood flow [23]. Additionally, previous research has confirmed the association between PA and sleep duration, and optimal sleep duration is frequently accompanied by up to standard exercise guidelines [23,24,25]. Therefore, based on the strong temporal association between sleep duration, PA, and stroke risk, PA might be one of the underlying mechanisms in the relationship between average sleep duration and stroke risk.

However, the correlation between average sleep duration and stroke risk remains inconsistent. Only a few studies have observed that PA is simultaneously associated with average sleep duration and stroke risk. The purpose of this study was to explore the relationship between average sleep duration and stroke risk in normal Chinese suburbanites, as well as the mediating effects of PA, including activity intensity, time, and frequency.

## 2. Materials and Methods

### 2.1. Participants

This cross-sectional study was conducted in the Fengxian District, a representative suburban area of Shanghai, from December 2018 to April 2019. The target population was the residents who had lived in the Fengxian District, Shanghai for more than 6 months, aged over 30 years (30~80 years), without a history of stroke or transient ischemic attack (TIA). People who did not live in this area and those who refused to participate or complete and sign the informed consent form were excluded. People with difficulty in communication due to serious physical or mental illness were also excluded. This study was approved by the Ethics Review Committee of the Shanghai University of Medicine & Health Sciences. Participation in this study was anonymous and voluntary. All participants signed a consent form to authorize the data collection before the start of the study. They were also informed of its purpose and the possibility of participation and withdrawal at any stage of the trial.

### 2.2. Instruments and Measurements

#### 2.2.1. Self-Made Questionnaire

Data on the sociodemographic characteristics of the inhabitants were collected using a multidimensional and self-made questionnaire that was completed by the patients to determine their gender, age, education, marital status, employment, smoking habit, drinking habit, height (m), and weight (kg). The body mass index (BMI) was calculated as weight/height^2^ (kg/m^2^), and a BMI of ≥26 kg/m^2^ was defined as obviously overweight or obese according to the stroke risk assessment form.

#### 2.2.2. Stroke Risk

According to the risk assessment form for a high-risk stroke population developed by the Stroke Prevention and Treatment Engineering Committee of the National Health and Family Planning Commission of China [26], the following eight risk factors were screened: (1) hypertension (a history of high blood pressure of ≥140/90 mmHg reported by the participants or diagnosed by physicians) or antihypertensive medication use; (2) heart disease (diagnosed by physicians), such as atrial fibrillation and/or valvular heart disease; (3) smoking (continuous or cumulative use of any tobacco product for more than 6 months over one’s lifetime); (4) dyslipidemia (diagnosed by physicians); (5) diabetes (diabetes mellitus, as defined by a prior diagnosis, a fasting plasma glucose level of ≥7.0 mmol/L, or a random blood glucose level of ≥11.0 mmol/L); (6) physical inactivity (physical exercise was defined as 30 min of physical activity over three times a week for more than a year, according to the National Physical Fitness Monitoring Program of 2000; physical labor in agriculture was also considered as physical activity; thus, the others were classified as physical inactivity); (7) Apparently overweight or obese (BMI ≥26 kg/m^2^); (8) family history of stroke. The stroke risk factors were graded as follows: high-risk group, with any 3 or more risk factors from the above 8 conditions; medium-risk group, suffering from chronic diseases (hypertension, diabetes, and/or heart disease) but with less than 3 risk factors; low-risk group, with less than 3 risk factors and no chronic diseases.

#### 2.2.3. Sleep Durations

The participants were questioned via a questionnaire about their night sleep duration (the period between going to bed at night and waking up in the morning except for awake time) and afternoon nap duration during the previous month to determine their average sleep duration. The total of the two sessions was the average sleep duration. The average sleeping duration was grouped as category 2 for 7~8 h per day, which was considered the control group—a relatively healthier sleeping duration according to the National Sleep Foundation [27]—and as categories 1, 3, and 4 for <7 h per day, >8~9 h per day, and >9 h per day, respectively. The division of long sleep duration (>8~9 h & >9 h) can further study the relationship between excessive sleep duration (>9 h) and stroke risk.

#### 2.2.4. Physical Activity

Physical activity in the last month was assessed using the Physical Activity Rating Scale-3 (PARS-3), which was compiled by the Japanese psychologist Gongxiong Hashimoto and modified into a Chinese version by Liang Deqing of the Wuhan Sports University [28]. The 3-item self-reported scale comprised of intensity, time, and frequency, as well as a 5-point Likert scale, was used for quantification, with each item scored from 1 to 5 points, and measured the level of physical activity involvement. The following formula was used to calculate the total score of physical activity: intensity × (time − 1) × frequency, with a score range of 0 to 100. Physical activity levels were classified as low-intensity (≤19 points), moderate-intensity (20~42 points), and high-intensity (≥43 points) based on the score. With a test–retest reliability of 0.82 and an internal consistency coefficient of 0.75, the scale exhibited a high reliability and validity in Chinese individuals [29].

### 2.3. Data Collection

Permission from the villages authorities was obtained to access their residences for the data collection and for residents to participate. The interviewers were medical students who received a two-week uniform formal training for conducting the interviews before the formal study. They were accompanied by the village cadres to visit each household, explaining the purpose and scope of the research, and instructed literate residents on how to complete the questionnaires, while illiterate individuals were interviewed face-to-face. The data analyst as a quality control person would then select 5% of the participants for repeat surveys and excluded the questionnaires with major discrepancies as dropouts to ensure the consistency, accuracy, and integrity of the questionnaire.

### 2.4. Sampling Method

**Figure 1:**
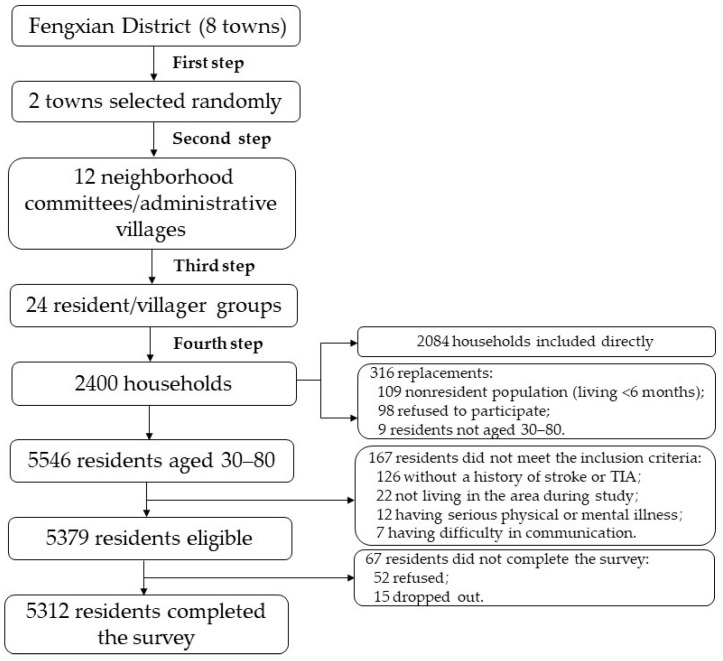
Flowchart of sampling method.

A multistage, stratified, probability-proportional-to-size sampling was adopted to identify participants. Two towns were selected randomly from eight towns in the Fengxian District in the first stage. Six neighborhood committees/administrative villages were selected at random from each town in the second stage. In the third step, each neighborhood committee/administrative village picked two resident/villager groups consisting of more than 100 households at random. In the fourth step, 100 households were picked at random from each resident/villager group. One to three participants per household were surveyed. If the selected households did not match the inclusion criteria or refused to be surveyed in the field, they were replaced. During the replacement method, residents from the same resident/villager group as the investigation households or from neighboring resident/villager groups were chosen based on the principles of living nearby and having a comparable family structure. In the end, 229 households failed to fulfill the inclusion requirements, and 87 households declined to participate in the survey, resulting in 316 replacements, a replacement rate of 13.17% (Figure 1).

### 2.5. Statistical Analysis

The data collected were entered and analyzed by SPSS (Statistical Package for Social Sciences) version 25.0 for Windows. The Kolmogorov–Smirnov test was used to determine the normality of continuous variables. The mean with standard deviation (SD) was used to describe variables that conformed to a normal distribution, whereas the median or interquartile range (IQR) was used to represent variables that did not follow a normal distribution. The categorical variables were stated as the frequency and percentage. Continuous variables were analyzed using a one-way ANOVA and the S–N–K test, while categorical variables were analyzed using Pearson’s chi-square.

All analyses were adjusted for sex, age, education, marital status, employment, and drinking. After adjusting for all covariables, we utilized multiple linear regression to identify the association between stroke risk, average sleep duration, and physical activity (PA). A mediation analysis was used to examine the role of the mediating variable (M) in the association between the independent variable (X) and the outcome variable (Y). The PROCESS macro v.3.5, authored by Andrew F Hayes, was used to create the mediation model. The dependent variable was stroke risk, the mediating factors were physical activity such as intensity, time, and frequency, and the independent variable was the average sleep duration. The total effect (path c) of average sleep duration on stroke risk was the sum of the direct and indirect effects. The direct effect (path c′) was the effect of average sleep duration on stroke risk after the adjustment for physical activity. The indirect effect (path ab) was the mediating impact of the link between average sleep duration and stroke risk. In addition, the three components of physical activity were examined independently as mediating factors to see if activity intensity, time, and frequency had mediation effects on average sleep duration and stroke risk. We employed the bootstrap method of the PROCESS macro to confirm the statistical significance of the mediating effect. The indirect effect (mediation effect) was estimated by a 95% confidence interval through 1000 bias-corrected bootstrap samples. We used the 95% CI excluding zero to determine if the mediating effects were statistically significant, as advised by Hayes. A *p*-value of <0.05 was considered statistically significant.

## 3. Results

### 3.1. Subject Characteristics and Differences

The study comprised 5312 participants (2342 men and 2970 women), with a mean age of 54.96 ± 12.21 years. Table 1 shows the sociodemographic characteristics of the individuals in our research. Significant differences were detected in different sleep duration groups excepting the marital status among the characteristics studied. Table 2 displays the compositions of the eight risk factors for stroke among the varied sleep duration groups. Only a family history of stroke had no significant difference among the four groups. For physical activity, various sleep duration groups had significant differences in levels, activity intensity, time, and frequency, which are showed in Table 3.

### 3.2. Relationship between Mean Sleep Time and Stroke Risk

Table 4 shows the association between average sleep duration and stroke risk in adults without a stroke or TIA using a multivariate linear regression analysis. In general, stroke risk was independently significantly associated with sleep duration and PA. After the adjustment for sex, age, education, marital status, employment, and drinking, a somewhat unhealthier sleeping duration (<7 h/>8 h) was significantly positively associated with stroke risk (β = 0.038, 95% CI: 0.024~0.128; β = 0.078, 95% CI: 0.128~0.250; β = 0.150, 95% CI: 0.390~0.549), compared with the moderate average sleep duration (7~8 h). This implied that an unhealthier sleep duration was linked to a greater risk of stroke. In the adjusted model, in addition to exercise intensity, activity duration (β = −0.105; 95% CI: −0.083~−0.046) and activity frequency (β = −0.126, 95% CI: −0.108~−0.063) were negatively correlated with stroke risk, suggesting that activity intensity was not associated with stroke risk, and a prolonged activity duration and frequency could reduce stroke risk.

### 3.3. Mediating Effects of Physical Activity on Average Sleep Duration and Stroke Risk

**Figure 2:**
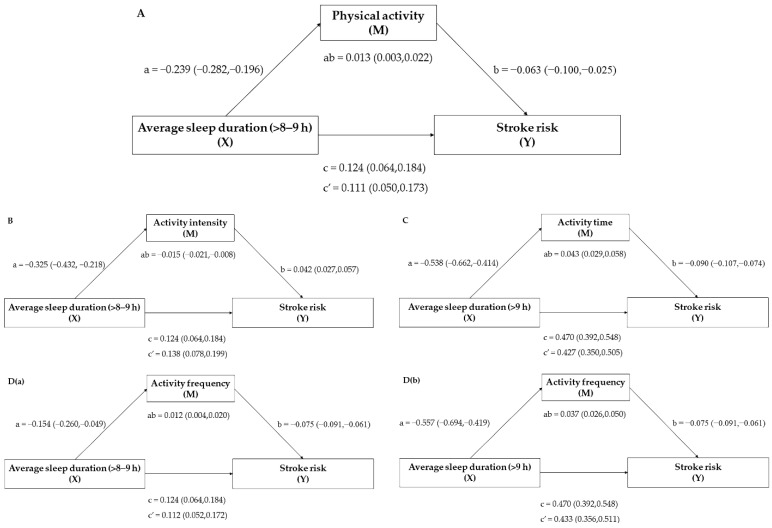
Mediation model (**A**) for the relationship between a sleep duration of >8~9 h and stroke risk, mediated by physical activity total score; (**B**) for the relationship between a sleep duration of >8~9 h and stroke risk, mediated by activity intensity; (**C**) for the relationship between a sleep duration of >9 h and stroke risk, mediated by activity duration; (**D**(**a**)) for the relationship between a sleep duration of >8~9 h and stroke risk, mediated by activity frequency; and (**D**(**b**)) for the relationship between a sleep duration of >9 h and stroke risk, mediated by activity frequency. Paths a, b, c, and c′ are presented as unstandardized coefficients (95% CI). a = X on M; b = M on Y; c = total effect of X on Y; c′ = direct effect of X on Y; ab = indirect effect of X on Y. Covariates: sex, age, education, marital status, employment, and drinking.

The mediating effect of physical activity on the relationship between the average sleep duration and the stroke risk is depicted in Table 5 and Figure 2A. Sleep duration of >8~9 h was shown to be strongly related to stroke risk via the physical activity total score (direct effect c′ = 0.111, 95% CI: 0.050~0.173), with the physical activity total score acting as a moderator (indirect effect ab = 0.013, 95%CI: 0.003~0.022). Although the sleep duration of >9 h was also substantially linked with stroke risk after adjusting for physical activity (direct effect c′ = 0.461, 95% CI: 0.382~0.540), the physical activity total score had no mediation influence (indirect effect ab = 0.009, 95% CI: −0.003~0.022).

The mediating effects of individual physical activity components were further analyzed. Table 6 and Figure 2B–D show the mediating effects of activity intensity, activity duration, and activity frequency on average sleep duration and stroke risk. For physical activity, although there was a significant correlation between an average sleep duration of >8~9 h (direct effect c′ = 0.138, 95% CI: 0.078~0.199) and >9 h (direct effect c′ = 0.473, 95%CI: 0.395~0.551) and stroke risk after adjusting for activity intensity, the activity intensity only had a mediation influence (indirect effect ab = −0.015, 95% CI: −0.021~−0.008) on an average sleep duration of >8~9 h and stroke risk. Moreover, there was a significant association between an average sleep duration of >8~9 h (direct effect c′ = 0.122, 95% CI: 0.062~0.182) and >9 h (direct effect c′ = 0.427, 95% CI: 0.350~0.505) and stroke risk after controlling for the activity duration, while the activity duration only had a mediating effect (indirect effect ab = 0.0430, 95% CI: 0.029~0.058) on the average sleep duration of >9 h and stroke risk. In terms of activity frequency, it mediated the link between an average sleep duration of both >8~9 h (indirect effect ab = 0.012, 95% CI: 0.004~0.020) and >9 h (indirect effect ab = 0.037, 95% CI: 0.026~0.050) and stroke risk. In conclusion, the relationship between sleep duration and stroke risk, and the mediating role of PA were only found between a long sleep duration (>8 h) and stroke risk, with a positive association of longer sleep duration with greater stroke risk. For PA, the activity intensity and activity duration only mediated the risk of stroke in certain sleep durations (>8~9 h/>9 h), whereas the activity frequency mediated the risk of stroke in all long sleep durations (>8 h). Therefore, avoiding an excessively long sleep duration and increasing the physical activity frequency could be more effective in improving stroke risk compared with increasing the activity intensity and duration.

## 4. Discussion

This study revealed that comparatively, an inappropriate sleep duration (<7 h/>8 h) was significantly positively related to a higher risk of stroke, and the physical activity total score had a mediating effect after correcting for sex, age, education, marital status, employment, and drinking. A further analysis of the role of the physical activity components revealed that each component partially mediated the association between the long sleep duration and stroke risk, but they worked differently in different sleep durations; for example, physical activity intensity moderated the relationship between a sleep duration of >8~9 h and stroke risk, while activity duration was effective when the sleep duration was over 9 h, and exercise frequency had a role in the two longer sleep durations (>8~9 h & >9 h). To our knowledge, this was the first study to explore the role of physical activity in mediating the association between average sleep duration and stroke risk in China’s suburbs.

Although a sleep duration of more than 8 h was linked to a higher risk of stroke than a sleep duration of 7~8 h, the link between a sleep duration of >8~9 h and stroke risk was weaker than the link between a sleep duration of >9 h and stroke risk, and the link between a decreased sleep duration and stroke risk was the weakest. A long sleep duration was significantly related with a higher risk of stroke in a large prospective cohort analysis, whereas a short sleep duration had no statistically significant influence on stroke risk [12]. A review also suggested that sleep deprivation screening should be considered as part of community-based primary stroke prevention, and the importance of adequate sleep needs to be emphasized [9]. However, another follow-up study showed that both a decrement and an increment of the average sleep duration were linked to stroke risk factors, which was consistent with our findings [30]. This inconsistent relationship might be owing in part to the ambiguous criteria of long sleep and short sleep durations. However, a meta-analysis of pooled data from cohort studies found no causal relationship between sleep duration (defined both subjectively and objectively) and stroke risk [16], and a dose–response meta-analysis of prospective cohort studies found a slightly lower risk of ischemic stroke among short-duration sleepers [31], both of which were not supported by our findings.

Physical inactivity may be one of the more readily occurring risk factors for stroke. The PA is an essential primary preventive measure for lowering the incidence of stroke. In the adapted model, we found an association between PA and stroke risk, excepted for exercise intensity. According to a cohort study of California women teachers, concurrent physical exercise reduced the short-term increased stroke risk [32]. The result of the reanalysis of a meta-analysis revealed that those who exercise in their leisure time have a lower risk of stroke. However, the observations from small research studies showed that the findings may be overstated [33]. Our findings were also in line with the result of a previous scoping review that found that breaking up prolonged sitting with frequent physical activity or standing had a favorable effect on some stroke risk variables (hypertension and dysglycemia) in the population at risk of a stroke [21]. A narrative review, unlike our study, found that prestroke PA reduced the risk of stroke and that its intensity was a key factor, with higher intensities leading to greater benefits, including improved health; however, the optimal intensity of the PA and exercise was not clear, and more research is needed [20].

We included PA in the research of average sleep duration and stroke risk because earlier studies had found a strong link between a greater PA and a lower stroke risk [34,35]. Higher PA scores, activity duration, and frequency, as well as lower activity intensity levels, were associated with an elevated likelihood of stroke risk factors among long-duration sleepers, according to our findings. The PA and its components all significantly mediated the connection between a long sleep duration and stroke risk factors (Figure 2).

The PA total score was found to have a partial mediating influence on the link between a long sleep duration (>8~9 h) and stroke risk (Figure 2A). According to research conducted in Croatia, people with a short sleep duration were less likely to report sufficient PA, whereas those with a long sleep duration were more likely to report adequate PA [36]. This result was probably because a short sleep duration was linked to a lower maximal oxygen uptake, higher exercise-related injuries, and daytime weariness, all of which led to a drop in daily PA participation.

The effect of the PA components was investigated further, and all three were found to partially mitigate the association between a long sleep duration and stroke risk factors (Figure 2B–D). The majority of PA and stroke research has thus far concentrated on the effects of physical activity on poststroke rehabilitation [37,38,39,40]. Few studies have looked at the relationship between PA and risk variables in nonstroke patients, let alone clarified the link between its components (activity intensity, duration, and frequency) and stroke risk factors. Future research should focus on the mechanism by which PA intensity, duration, and frequency modulate the sleep duration and stroke risk factors, as well as the extent to which PA intensity, duration, and frequency can maximize the reduction of stroke risk factors.

Given that improving PA and sleep can significantly reduce cardiometabolic risk, activity intensity, duration, and frequency all played a mediating role in the relationship between long average sleep duration and stroke risk, albeit in different durations, and targeted interventions for exercise (intensity, duration, and frequency) and sleep duration may prevent stroke.

There are several limitations to this study as well. First, rather than objective measurements, self-reported questionnaires were used to collect data on average sleep duration and PA. Although self-reporting of sleep duration and PA has been one of the most often used approach, sleep duration and PA recorded by actigraphy-measured evaluations should be more reliable, yet objective sleep and PA data in large population studies are infeasible to collect. To improve the accuracy and authenticity of the data, the average sleep duration was calculated by asking the participants to recall the time they fell asleep at night, the period they kept awake at night, the time they woke up in the morning and the duration they napped during the day, instead of asking them to answer directly. PA was assessed using the PARS-3 with a high reliability and validity. In addition, we repeated the survey with 5% of the participants, and questions with major discrepancies in the results were excluded from the data gathering process to improve the data’s authenticity. Second, associated sleep disorders including snoring, night-awakening, apnea, and somnolence may also inevitably have an impact on sleep duration to some extent. Third, because our study was cross-sectional, we were unable to verify causation; nevertheless, a mediation analysis might be used to investigate potential pathways based on association.

## 5. Conclusions

Our research found a significant link between a long average sleep duration and stroke risk among Chinese suburban residents without a stroke, when compared to a moderate average sleep duration. The PA and its components were found to mediate the link between a long average sleep duration and the risk of stroke. Furthermore, compared with activity intensity and activity duration, increasing activity frequency could be more effective at improving stroke risk. This finding reveals that the PA could be a potential mechanism between average sleep duration and stroke risk, and that an adequate exercise intensity, duration, and frequency, as well as sleep interventions, may help prevent future strokes by reducing risk factors.

## Figures and Tables

**Table 1:** Sociodemographic characteristics across categories of average sleep duration.

Variable		Sleep	Duration (h)		*p*
	<7 (*n* = 941)	7~8 (*n* = 3603)	>8~9 (*n* = 540)	>9 (*n* = 228)	
Gender					**<0.001**
Male	356 (15.2%)	1513 (64.6%)	287 (12.3%)	186 (7.9%)	
Female	553 (18.6%)	1958 (65.9%)	309 (10.4%)	150 (5.1%)	
Age (years)	58.66 ± 11.22	53.68 ± 12.18	55.45 ± 12.34	57.29 ± 12.40	**<0.001**
≤45	142 (10.6%)	997 (74.3%)	139 (10.4%)	64 (4.8%)	
46~55	184 (15.0%)	838 (68.4%)	136 (11.1%)	67 (5.5%)	
56~65	278 (18.5%)	943 (62.6%)	185 (12.3%)	100 (6.6%)	
≥66	305 (24.6%)	693 (55.9%)	136 (11.0%)	105 (8.5%)	
Education					**<0.001**
Primary and below	306 (33.7%)	688 (19.8%)	146 (24.5%)	103 (30.7%)	
Middle and high school	391 (43.0%)	1646 (47.4%)	306 (51.3%)	173 (51.5%)	
College and above	212 (23.3%)	1137 (32.8%)	144 (24.2%)	60 (17.9%)	
Marital status					0.319
With partner	825 (16.9%)	3205 (65.6%)	550 (11.3%)	304 (6.2%)	
Single/widow/separated	84 (19.6%)	266 (62.1%)	46 (10.7%)	32 (7.5%)	
Employment					**<0.001**
Stable	321 (14.1%)	1582 (69.4%)	252 (11.1%)	123 (5.4%)	
Unstable	588 (19.4%)	1889 (62.3%)	344 (11.3%)	213 (7.0%)	

Significant effects are denoted in bold.

**Table 2:** Stroke risk factors across categories of average sleep duration.

Variable		Sleep	Duration (h)		*p*
	<7 (*n* = 941)	7~8 (*n* = 3603)	>8~9 (*n* = 540)	>9 (*n* = 228)	
Hypertension					**<0.001**
Yes	285 (21.8%)	706 (54.1%)	177 (13.6%)	137 (10.5%)	
no	624 (15.6%)	2765 (69.0%)	419 (10.5%)	199 (5.0%)	
Dyslipidemia					**<0.001**
Yes	113 (20.1%)	300 (53.4%)	83 (14.8%)	66 (11.7%)	
no	796 (16.8%)	3171 (66.8%)	513 (10.8%)	270 (5.7%)	
Diabetes					**<0.001**
Yes	77 (19.3%)	213 (53.3%)	54 (13.5%)	56 (14.0%)	
no	832 (16.9%)	3258 (66.3%)	542 (11.0%)	280 (5.7%)	
Atrial fibrillation					
Yes	69 (21.0%)	175 (53.4%)	42 (12.8%)	42 (12.8%)	**<0.001**
no	840 (16.9%)	3296 (66.1%)	554 (11.1%)	294 (5.9%)	
Smoking					**<0.001**
Yes	172 (14.7%)	702 (59.9%)	164 (14.0%)	133 (11.4%)	
no	737 (17.8%)	2769 (66.9%)	432 (10.4%)	203 (4.9%)	
Overweight	23.76 ± 3.47	23.54 ± 3.33	23.75 ± 3.20	24.58 ± 3.56	**<0.001**
Yes	65 (16.8%)	218 (56.5%)	53 (13.7%)	50 (13.0%)	
No	844 (17.1%)	3253 (66.0%)	543 (11.0%)	286 (5.8%)	
Physical inactivity					**<0.001**
Yes	535 (16.5%)	2120 (65.3%)	350 (10.8%)	244 (7.5%)	
no	374 (18.1%)	1351 (65.5%)	246 (11.9%)	92 (4.5%)	
Family history of stroke					0.294
Yes	79 (18.8%)	279 (66.4%)	36 (8.6%)	26 (6.2%)	
no	830 (17.0%)	3192 (65.2%)	560 (11.4%)	310 (6.3%)	

Significant effects are denoted in bold.

**Table 3:** Physical activity across categories of average sleep duration.

Variable		Sleep	Duration (h)		*p*
	<7 (*n* = 941)	7~8 (*n* = 3603)	>8~9 (*n* = 540)	>9 (*n* = 228)	
Physical activity score	12.59 ± 10.32	16.16 ± 9.13	12.34 ± 8.82	9.47 ± 8.46	**<0.001**
low intensity	723 (20.7%)	1932 (55.2%)	520 (14.9%)	324 (9.3%)	
moderate intensity	161 (9.3%)	1494 (86.3%)	69 (4.0%)	8 (0.5%)	
high intensity	25 (30.9%)	45 (55.6%)	7 (8.6%)	4 (4.9%)	
Activity intensity score	2.28 ± 1.28	2.49 ± 1.30	2.10 ± 1.10	2.32 ± 1.10	**<0.001**
Activity duration score	2.97 ± 1.17	3.26 ± 1.08	3.13 ± 1.14	2.63 ± 1.22	**<0.001**
Activity frequency score	3.11 ± 1.25	3.36 ± 1.24	3.12 ± 1.21	2.74 ± 1.11	**<0.001**

Significant effects are denoted in bold.

**Table 4:** Associations of stroke risk with average sleep duration and physical activity by a multiple linear regression analysis.

	*β* Coefficient (95%CI)			
Variables	Unadjusted Model	*p*	Adjusted Model	*p*
Average sleep duration				
<7 h	0.104 (0.100~0.210)	**<0.001**	0.038 (0.024~0.128)	**0.04**
7~8 h (Ref.)				
>8~9 h	0.088 (0.168~0.299)	**<0.001**	0.078 (0.128~0.250)	**<0.001**
>9 h	0.191 (0.502~0.672)	**<0.001**	0.150 (0.390~0.549)	**<0.001**
Physical activity	0.080 (0.063~0.178)	**<0.001**	0.092 (0.084~0.191)	**<0.001**
Activity intensity	−0.056 (−0.058~−0.013)	**0.002**	−0.034 (−0.042~0.001)	0.063
Activity duration	−0.084 (−0.072~−0.033)	**<0.001**	−0.105 (−0.083~−0.046)	**<0.001**
Activity frequency	−0.136 (−0.119~−0.072)	**<0.001**	−0.126 (−0.108~−0.063)	**<0.001**

Significant effects are denoted in bold.

**Table 5:** Summary of the mediation analysis for average sleep duration, physical activity, and stroke risk.

Independent Variable	Mediating Variable	Dependent Variable	Coefficient (Bias-Corrected Bootstrap 95% CI)		
Average sleep duration	Physical activity	Stroke risk	Indirect effect (ab)	Total effect (c)	Direct effect (c′)
<7 h			**0.008 (0.003~0.014)**	0.026 (−0.025~0.077)	0.018 (−0.034~0.069)
7~8 h (Ref.)					
>8~9 h			**0.013 (0.003~0.022)**	**0.124 (0.064~0.184)**	**0.111 (0.050~0.173)**
>9 h			0.009 (−0.003~0.022)	**0.470 (0.392~0.548)**	**0.461 (0.382~0.540)**

Significant effects are denoted in bold.

**Table 6:** Summary of the mediation analysis for average sleep duration, physical activity components, and stroke risk.

Independent Variable	Mediating Variable	Dependent Variable	Coefficient (Bias-Corrected Bootstrap 95% CI)		
Average Sleep Duration	Physical Activity	Stroke Risk	Indirect Effect (ab)	Total Effect (c)	Direct Effect (c′)
	Activity intensity				
<7 h			−0.004 (−0.008~0.0001)	0.026 (−0.025~0.077)	0.030 (−0.022~0.081)
7~8 h (Ref.)					
>8~9 h			**−0.015 (−0.021~−0.008)**	**0.124 (0.064~0.184)**	**0.138 (0.078~0.199)**
>9 h			−0.003 (−0.009~0.003)	**0.470 (0.392~0.548)**	**0.473 (0.395~0.551)**
	Activity duration				
<7 h			**0.020 (0.012~0.028)**	0.026 (−0.025~0.077)	0.006 (−0.045~0.057)
7~8 h (Ref.)					
>8~9 h			0.117 (−0.007~0.011)	**0.124 (0.064~0.184)**	**0.122 (0.062~0.182)**
>9 h			**0.043 (0.029~0.058)**	**0.470 (0.392~0.548)**	**0.427 (0.350~0.505)**
	Activity frequency				
<7 h			**0.015 (0.008~0.023)**	0.026 (−0.025~0.077)	0.011 (−0.040~0.062)
7~8 h (Ref.)					
>8~9 h			**0.012 (0.004~0.020)**	**0.124 (0.064~0.184)**	**0.112 (0.052~0.172** **)**
>9 h			**0.037 (0.026~0.050)**	**0.470 (0.392~0.548)**	**0.433 (0.356~0.511)**

Significant effects are denoted in bold. Covariates: sex, age, education, marital status, employment, and drinking.

## Data Availability

The datasets used and/or analyzed during the current study are available from the corresponding author on reasonable request.
